# Association Between Dairy Intake and Executive Function in Chinese Children Aged 6–12 Years

**DOI:** 10.3389/fnut.2022.879363

**Published:** 2022-07-11

**Authors:** Xia Zeng, Li Cai, Zhaohuan Gui, Tianran Shen, Wenhan Yang, Qingsong Chen, Yajun Chen

**Affiliations:** ^1^Department of Child and Adolescent Health, School of Public Health, Guangdong Pharmaceutical University, Guangzhou, China; ^2^Department of Maternal and Child Health, School of Public Health, Sun Yat-sen University, Guangzhou, China; ^3^Guangdong Provincial Engineering Research Center of Public Health Detection and Assessment, Guangdong Pharmaceutical University, Guangzhou, China

**Keywords:** children, dairy intake types, executive function, BRIEF-parent version, cross-sectional study

## Abstract

Association between dairy intake and executive function remains controversial, especially among children, a population with fast-developing executive functions. This study aimed to explore this topic. Additionally, we further distinguished the role of dairy intake types (full- or low-fat milk or yogurt) in this relationship. This survey included 5,138 children aged 6–12 years. Dairy intakes were assessed by validated questionnaires. Executive function was measured by the behavior rating inventory of executive function (BRIEF; Parent Version), and lower T-scores of BRIEF indices indicated superior executive function performance. Results showed that children with higher dairy intake had statistically better performance in Shift (46.58 ± 7.48 vs. 45.85 ± 7.10), Initiate (48.02 ± 8.58 vs. 47.14 ± 8.33), and Working Memory (50.69 ± 8.82 vs. 49.89 ± 8.73). In the analysis of multivariate linear regression, we found that for every one unit increase in full-fat dairy intake, T-scores for Shift (β = −0.350 (95% confidence interval [CI]: (−0.660, −0.039) and Initiate (β = −0.486 (95% CI: (−0.845, −0.127) were decreased and for every one unit increase in low-fat dairy intake, T-score for Organizations of Materials (β = −0.940 (95% CI: (−1.690, −0.189) was decreased. After distinguishing dairy into milk and yogurt, we observed that only milk intake, not yogurt, was significantly associated with better executive function performance in Shift (β = −0.390 (95% CI (−0.745, −0.035) and Initiate (β = −0.509 (95% CI (−0.917, −0.101) after adjusting for potential confounding factors. This study shows that a higher intake of dairy, irrespective of fat content, is related to better executive function performance among children aged 6–12. In addition, a significantly positive relationship between dairy intake and executive function’s indices of Shift and Initiate only was observed in milk, not in yogurt.

## Introduction

Executive function, a particularly crucial domain within cognitive processing, consists of the mental capacity to make goal-directed behaviors, including inhibitory control, working memory, attention, and planning (1). Executive function skills develop significantly throughout childhood, with ongoing maturation continuing into adolescents and adulthood (2). A higher level of executive function plays an important role in both academic achievement (3) and health-related decision-making among children and adolescents (4–6). Thus, identifying modifiable factors, such as dairy intake, is generally considered to play an important role in children’s executive function development.

A milk-derived tripeptide, Tyr-Leu-Gly (YLG), has been shown in animal experiments to contribute to executive function development by promoting the growth of nerve growth factor (NGF) and glial cell line-derived neurotrophic factor in the hippocampus (7). Milk is a great source of high-quality protein, minerals, vitamins, insulin-like growth factors, polyunsaturated fatty acids, and bioactive peptides (8). Previous studies have documented that more milk intake was beneficial to childhood growth and physical health (8, 9). Evidence shows that dairy intake, which includes liquid milk, such as milk and yogurt, is associated with executive function in the elderly (10–14). Unlike elderly individuals whose cognitive level is declined, children are in a stage of rapid development of executive functions (15). The difference in the development process of executive function implies that associations of dairy intake with executive function among the elderly may not be extrapolated to children. It has become a public consensus that dairy intake can promote the physical development of children and adolescents (16), but studies investigating the association between dairy intake and executive function in children are still limited and controversial (9, 17–20). Two school-based trials have shown that children who drink milk or more milk have better learning and executive function performance than those with no milk or less milk (9, 20), although the studies did not distinguish types of dairy intake, such as full- or low-fat milk or yogurt. Inconsistently, results of a review reported that only one-third of studies agree that milk intake would affect the cognitive function-related abilities (intelligence, academic performance, etc.) of school-age children (19). Studies conducted among adults indicated that the impacts of dairy intake on cardiovascular and metabolic health are mainly due to sugar content and not just fat content (21, 22). Yogurt sold on the market contains more sugar than regular milk (23). Based on this, we speculate that the possible reason for the inconsistent relationship between dairy intake and children’s executive function is different types of dairy intake. However, as far as we know, there is still a lack of direct evidence that distinguishes dairy intake from milk and yogurt to explore their relationship with children’s executive function.

Therefore, there is a need for an extensive and comprehensive assessment of the impact of dairy intake on executive function among children to help better inform future recommendations regarding dairy intake and developing executive function among children. In the present study, we hypothesize that those with higher dairy intake will have better executive function performance, and this relationship varies depending on the types of dairy intake.

## Materials and Methods

### Study Design and Participants

**FIGURE 1:**
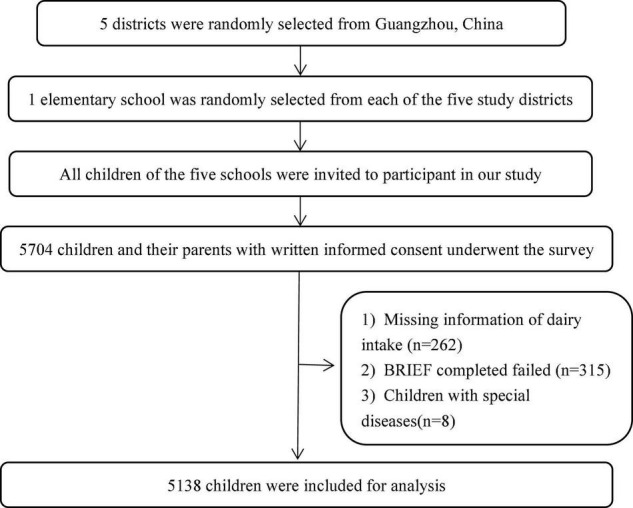
Flowchart of participants’ inclusion.

The data were from the baseline examination of a school-based prospective cohort study (Clinical Trial Registration Number: NCT03582709). This study was approved by the Ethics and Human Subject Committee of Sun Yat-sen University, and the design has been described in detail elsewhere (24). Briefly, the multistage stratified random cluster sampling method was adopted to recruit study participants from March to May 2017. First, we randomly selected five districts from Guangzhou city. Second, we randomly selected one elementary school from each study district. All children of the five schools were invited to participate and complete the questionnaire. Children who were younger than 6 years or older than 12 years and diagnosed with visceral diseases, abnormal growth and development, or mental health problems were excluded from the study. All children and their parents voluntarily participated in this project with informed consent forms. The final sample consisted of 5,138 children and their parents completed questionnaires and anthropometric measurements, and the response rate was 90.1% (5,138/5,704). We excluded those who had missing information about dairy intake (*n* = 262) or on the behavior rating inventory of executive function (BRIEF; *n* = 315) and children with diseases (*n* = 8; Figure 1).

### Questionnaire Survey

#### Dairy Intake

A validated self-reported questionnaire, completed by children and their parents together, was used to assess the consumption of dairy (25). The questions were put forth as: “In the past 7 days, how many times did you consume dairy products in total (such as full-, low-, and skimmed-fat milk, yogurt, or milk powder)? and how many milliliters (ml) of them did you consume each time?” (1 box of milk is equivalent to 250 ml, 1 bottle of yogurt is equivalent to 150 ml, and 1 scoop of milk powder is equivalent to 30 ml). In order to obtain information on dairy intake of different types, we further investigated separately the frequency and quantity of full-, low-, and skimmed-fat milk or yogurt intake in the past 7 days. The questions were as follows: “How many times did you drink full-, low-, and skimmed-fat milk and yogurt during the days you consumed dairy products? How many milliliters did you drink each time?” Finally, we calculated the total milliliters of daily dairy intake and the daily dairy intake of full-, low-, and skimmed-fat milk and yogurt, respectively. In the analysis, we combined skimmed-fat dairy and low-fat dairy into the group of low-fat dairy intake. Full-fat dairy includes full-fat milk and full-fat yogurt, low-fat dairy includes low-fat milk and low-fat yogurt, milk includes full- and low-fat milk, and yogurt includes full- and low-fat yogurt.

#### Executive Function

We used the parents-rated BRIEF to assess children’s executive function during the past 6 months, which is widely used in epidemiological studies (26, 27). The BRIEF is an 86-item report with a 4-point response scale (response for each is “No” [1 point]), “Sometimes” [2 points], or “Often” [3 points]) (28). It contains eight clinical domains (Inhibit, Shift, Emotional Control, Initiate, Working Memory, Plan/Organize, Organization of Materials, and Monitor) that comprise two index scores: Behavioral Regulation Index (BRI) and Metacognition Index (MI). Inhibit, Shift, and Emotional Control constitute the BRI, with the remaining domains constituting the MI. BRI and MI combine to form the global executive function score (GEC). Supplementary Table 1 shows the detailed description of the indicator of the BRIEF scale. The T-scores of the BRIEF were adjusted for age and sex according to previously published normative values (27). Higher T-score indicates greater degrees of executive dysfunction. T-scores of 65 or higher are considered “clinically increased” levels. This inventory is confirmed to have a good internal consistency of 0.74–0.96, a good convergent validity of 0.41–0.64, and a good test-retest of 0.68–0.89 in school-aged children in China (29) and other countries (30) according to previous studies.

### Assessment of Covariates

The physical examination included height and weight, which were tested by professionals. Body mass index (BMI) was calculated as weight in kilograms divided by height in meters squared. Sociodemographic factors (e.g., children’s birth date, sex, paternal and maternal educational level, and household monthly income) were reported by parents. Paternal and maternal educational level was categorized into three groups (high school or below, junior college, and college or above). Monthly household income was divided into five groups (<5,000, 5,000–7,999, 8,000–11,999, ≥ 12,000, and “No answer”). Physical activities and sedentary time were reported by children and their parents according to the International Physical Activity Questionnaire Short Form. For other dietary behaviors, including the consumption of fruit, vegetable, cereals, fish, and red meat, children and their parents were required to report data on the frequency and quantity in the past 7 days, and the average daily food consumption was calculated. We obtained information about fried food through a question: “In the past 7 days, how many times have you eaten fried food?” Regarding sugar-sweetened beverages, we asked children the number of times and cups of sugar-sweetened beverages in the past 7 days and calculated the average daily amounts of sugar-sweetened beverages.

### Statistical Analysis

Continuous variables were reported as the mean ± standard deviation (SD), whereas categorical variables were shown as numbers and percentages. Children were divided into subgroups based on whether their daily dairy intake reached 300 ml. The differences in participant characteristics among dairy intake groups were compared using independent groups t-tests for continuous variables and Pearson’s chi-squared tests for categorical variables. Multivariate linear regression was carried out to evaluate the associations between dairy intake and executive function. Model 1 was adjusted for age, gender, paternal and maternal educational level, household monthly income, and BMI. Model 2 was further adjusted for moderate-to-vigorous intensity physical activity and after-school sedentary time. Model 3 was additionally adjusted for other dietary intakes, including fruits, vegetables, cereals, fish, red meat, fried food, and sugar-sweetened beverages. All the variables were entered simultaneously into multivariate linear models. All analyses were conducted using SPSS version 21.0 (IBM, Armonk, NY, United States) and a 2-sided *p* < 0.05 indicated statistical significance.

## Results

The analytic sample included 5,138 children, with a mean (SD = 1.73) age was 9.08 years and 2,706 (52.7%) were boys. The average children’s daily dairy intake is 180.81 ± 123.36 ml, of which 113.10 ± 115.75 ml was full-fat dairy intake and 27.66 ± 60.58 ml was low-fat dairy intake. Daily intake of milk and yogurt is 89.66 ± 103.35 ml and 50.18 ± 65.83 ml, respectively. The characteristics of children according to dairy intake levels are summarized in Table 1. Children who consumed a higher level of dairy were older, had higher BMI, had parents with a higher level of education, and consumed more fruits, vegetables, red meat, and sugar-sweetened beverages (*p* < 0.001).

**TABLE 1:** Descriptive characteristics of study children by dairy intake.

	All (*n* = 5,138)	<300 ml/day (*n* = 4,506)	≥300 ml/day (*n* = 632)	*P*
*Age, years*	9.08 ± 1.73	9.06 ± 1.73	9.19 ± 1.75	0.03
*Gender*				<0.001
Boy	2,706 (52.7)	2,310 (51.3)	396 (62.7)	
Girl	2,432 (47.3)	2,196 (48.7)	236 (37.3)	
*Paternal educational level*				<0.001
High school or below	1,226 (24.2)	1,112 (25.1)	114 (18.3)	
Junior college	1,238 (24.5)	1,100 (24.8)	138 (22.2)	
College or above	2,596 (51.3)	2,225 (50.1)	371 (59.6)	
*Maternal educational level*				<0.001
High school or below	1,325 (26.2)	1,199 (27.0)	126 (20.2)	
Junior college	1,375 (27.2)	1,212 (27.3)	163 (26.2)	
College or above	2,360 (46.6)	2,026 (45.7)	334 (53.6)	
*Household monthly income*				0.144
<5,000, CNY	1,071 (21.2)	940 (21.2)	131 (21.2)	
5,000∼7,999, CNY	1,299 (25.8)	1,127 (25.5)	172 (27.9)	
8,000∼11,999, CNY	891 (17.7)	785 (17.7)	106 (17.2)	
≥12,000, CNY	1,088 (21.6)	946 (21.4)	142 (23.0)	
No answer	695 (13.8)	629 (14.2)	66 (10.7)	
*BMI*	17.05 ± 3.22	17.00 ± 3.22	17.23 ± 3.09	0.043
*MVPA, min/day*	43.49 ± 36.43	43.17 ± 28.92	45.09 ± 29.74	0.076
*After-school ST, min/day*	170.13 ± 82.88	170.44 ± 82.73	166.42 ± 81.47	0.163
*Fruits, servings/day*	2.21 ± 1.99	2.14 ± 1.93	2.62 ± 2.20	<0.001
*Vegetables, servings/day*	3.98 ± 3.63	3.86 ± 3.52	4.63 ± 4.08	<0.001
*Cereals, servings/day*	0.89 ± 1.21	0.87 ± 1.18	0.94 ± 1.24	0.064
*Fish, servings/day*	1.15 ± 1.50	1.13 ± 1.47	1.24 ± 1.57	0.04
*Red meat, servings/day*	2.27 ± 2.49	2.16 ± 2.38	2.82 ± 2.90	<0.001
*Fried food, times/day*	0.56 ± 0.74	0.55 ± 0.73	0.57 ± 0.76	0.4
*Sugar-sweetened beverages, cups/day*	0.17 ± 0.26	0.16 ± 0.25	0.18 ± 0.30	0.013
*dairy intake, ml/day*	180.81 ± 123.36	146.86 ± 83.57	420.60 ± 87.96	<0.001
Full-fat dairy	113.10 ± 115.75	100.65 ± 102.36	205.00 ± 156.21	<0.001
Low-fat dairy	27.66 ± 60.58	26.96 ± 58.83	33.38 ± 72.07	0.004
Milk	89.66 ± 103.35	80.13 ± 93.79	160.31 ± 138.75	<0.001
Yogurt	50.18 ± 65.83	46.61 ± 62.60	75.09 ± 79.66	<0.001

*CNY, Chinese yuan; BMI, body mass index; MVPA, moderate-to-vigorous intensity physical activity; after-school ST, after-school sedentary time.*

Table 2 presents the BRIEF scores across different dairy intake groups. The results showed that children with higher dairy intake had statistically lower T-scores of BRIEF for Shift (46.58 ± 7.48 vs. 45.85 ± 7.10), Initiate (48.02 ± 8.58 vs. 47.14 ± 8.33), and Working Memory (50.69 ± 8.82 vs. 49.89 ± 8.73). Preliminary analysis revealed a reduction of BRIEF indices in the significant association between dairy intake and executive function with increasing adjustment for covariates (Table 3). In the analysis of multivariate linear regression, we found that for every one unit (150 ml) increase in dairy intake, the T-scores for GEC (β = −0.308 (95% confidence interval [CI] (−0.618, −0.004)), Shift (β = −0.404 (95% CI (−0.672, −0.137), BRI (β = −0.278 (95% CI (−0.537, −0.020), and Initiate (β = −0.443 (95% CI (−0.751, −0.137) were decreased when we adjusted for potential confounding factors (Model 3).

**TABLE 2:** Performance of children with different dairy intake on the scale of the BRIEF.

Scale/Index	All	<300 ml/day	≥300 ml/day	*P*
Inhibit	46.67 ± 7.42	46.67 ± 7.39	46.45 ± 7.48	0.458
Shift	46.52 ± 7.46	46.58 ± 7.48	45.85 ± 7.10	0.017
Emotional control	43.22 ± 7.15	43.25 ± 7.12	42.84 ± 7.07	0.16
*BRI*	44.55 ± 7.23	44.57 ± 7.22	44.17 ± 7.09	0.182
Initiate	47.95 ± 8.57	48.02 ± 8.58	47.14 ± 8.33	0.013
Working memory	50.62 ± 8.81	50.69 ± 8.82	49.89 ± 8.73	0.028
Plan/organize	52.15 ± 9.64	52.16 ± 9.59	51.74 ± 10.45	0.409
Organization of materials	46.53 ± 9.03	46.49 ± 9.04	46.67 ± 8.91	0.621
Monitor	52.21 ± 10.36	52.26 ± 10.33	51.74 ± 10.45	0.220
*MI*	50.04 ± 9.48	50.07 ± 9.47	49.63 ± 9.45	0.269
*GEC(BRI + MI)*	47.83 ± 8.60	47.85 ± 8.58	47.42 ± 8.57	0.237

*BRI, Behavioral Regulation Index; MI, Metacognition Index; GEC, global executive function score.*

**TABLE 3:** Multivariate linear regression for analyzing associations of dairy intake with executive function.

	Dairy intake unstandardized β (95% CI)		
Scale/Index	Model 1	Model 2	Model 3
Inhibit	–0.220 (–0.470, 0.029)	–0.165 (–0.429, 0.100)	–0.165 (–0.429, 0.101)
Shift	–0.424 (–0.674, –0.174)**	–0.404 (–0.672, –0.136)*	–0.404 (–0.672, –0.137)*
Emotional control	–0.230 (–0.470, 0.010)	–0.241 (–0.499, 0.018)	–0.241 (–0.499, 0.019)
*BRI*	–0.295 (–0.539, –0.052)*	–0.278 (–0.537, –0.019)*	–0.278 (–0.537, –0.020)*
Initiate	–0.552 (–0.839, –0.265)**	–0.443 (–0.751, –0.136)*	–0.443 (–0.751, –0.137)*
Working memory	–0.333 (–0.630, –0.037)*	–0.221 (–0.534, 0.092)	–0.221 (–0.534, 0.093)
Plan/Organize	–0.333 (–0.654, –0.011)*	–0.301 (–0.641, 0.038)	–0.301 (–0.641, 0.039)
Organization of materials	–0.295 (–0.597, 0.007)	–0.196 (–0.521, 0.129)	–0.196 (–0.521, 0.130)
Monitor	–0.389 (–0.736, –0.041)*	–0.266 (–0.639, 0.108)	–0.266 (–0.639, 0.109)
*MI*	–0.423 (–0.746, –0.099)*	–0.322 (–0.663, 0.019)	–0.322 (–0.663, 0.020)
*GEC (BRI + MI)*	–0.391 (–0.686, –0.096)*	–0.308 (–0.618, –0.003)*	–0.308 (–0.618, –0.004)*

BRI, Behavioral Regulation Index; MI, Metacognition Index; GEC, global executive function score. Model 1 is adjusted for age, gender, paternal and maternal educational level, household monthly income, and BMI. Model 2 is further adjusted for moderate-to-vigorous intensity physical activity and after-school sedentary time plus variables in Model 1. Model 3 is additionally adjusted for other dietary intakes, such as fruits, vegetables, cereals, fish, red meat, fried food, and sugar-sweetened beverages plus variables in Model 2. *p < 0.05, **p < 0.001.

To clarify whether there are differences in the relationship between different types of dairy intakes and executive functions, we further analyzed the association of full- and low-fat dairy intake, milk, and yogurt with indices of executive function. Children who consumed more full-fat dairy had lower scores in Shift (β = −0.350 (95% CI (−0.660, −0.039) and Initiate (β = −0.486 (95% CI (−0.845, −0.127) after adjusting for covariates (Table 4). However, the intake of low-fat dairy was significantly negatively correlated with the T-score for Organizations of Materials (β = −0.940 (95% CI (−1.690, −0.189). After distinguishing dairy into milk and yogurt (Table 5), we observed that only milk intake, not yogurt, was significantly associated with better executive function performance in Shift (β = −0.390 (95% CI (−0.745, −0.035) and Initiate (β = −0.509 (95% CI (−0.917, −0.101). Furthermore, Supplementary Table 2 shows that the statistically significant negative associations between milk intake and the T-scores of BRIEF mainly existed in the type of full-fat milk (*p* < 0.05).

**TABLE 4:** Multivariate linear regression for analyzing associations of full- or low-fat dairy intake with executive function.

	Full-fat dairy unstandardized β (95% CI)			Low-fat dairy unstandardized β (95% CI)		
Scale/Index	Model 1	Model 2	Model 3	Model 1	Model 2	Model 3
Inhibit	–0.240 (–0.513, 0.033)	–0.158 (–0.448, 0.131)	–0.188 (–0.494, 0.118)	0.002 (–0.527, 0.532)	0.081 (–0.481, 0.643)	0.204 (–0.404, 0.812)
Shift	–0.386 (–0.662, –0.110)*	–0.358 (–0.652, –0.064)*	–0.350 (–0.660, –0.039)*	–0.426 (–0.961, 0.110)	–0.299 (–0.869, 0.271)	–0.110 (–0.727, 0.506)
Emotional control	–0.199 (–0.463, 0.065)	–0.110 (–0.392, 0.172)	–0.117 (–0.417, 0.182)	–0.137 (–0.644, 0.370)	–0.252 (–0.795, 0.292)	–0.143 (–0.734, 0.448)
*BRI*	–0.296 (–0.564, –0.028)*	–0.221 (–0.505, 0.064)	–0.231 (–0.532, 0.069)	–0.157 (–0.677, 0.362)	–0.164 (–0.716, 0.387)	0.010 (–0.587, 0.606)
Initiate	–0.605 (–0.923, –0.287)**	–0.497 (–0.836, –0.157)*	–0.486 (–0.845, –0.127)*	–0.525 (–1.135, 0.085)	–0.259 (–0.911, 0.394)	–0.020 (–0.728, 0.688)
Working memory	–0.343 (–0.670, –0.016)*	–0.150 (–0.493, 0.194)	–0.134 (–0.496, 0.229)	–0.352 (–0.985, 0.280)	–0.209 (–0.874, 0.457)	–0.050 (–0.766, 0.665)
Plan/Organize	–0.322 (–0.679, 0.035)	–0.138 (–0.513, 0.236)	–0.096 (–0.492, 0.300)	–0.387 (–1.080, 0.305)	–0.155 (–0.884, 0.573)	–0.017 (–0.802, 0.767)
Organization of materials	–0.260 (–0.594, 0.074)	–0.082 (–0.440, 0.277))	–0.086 (–0.465, 0.294)	–0.989 (–1.633, –0.345)*	–0.884 (–1.576, –0.192)*	–0.940 (–1.690, –0.189)*
Monitor	–0.279 (–0.665, 0.107)	–0.062 (–0.473, 0.350)	–0.001 (–0.437, 0.436)	–0.540 (–1.283, 0.203)	–0.388 (–1.818, 0.406)	–0.221 (–1.083, 0.641)
*MI*	–0.340 (–0.699, 0.019)	–0.130 (–0.508, 0.248)	–0.093 (–0.491, 0.305)	–0.707 (–1.402, –0.011)*	–0.499 (–1.233, 0.236)	–0.370 (–1.155, 0.415)
*GEC (BRI + MI)*	–0.327 (–0.655, –0.001)*	–0.148 (–0.492, 0.195)	–0.130 (–0.492, 0.232)	–0.456 (–1.093, 0.181)	–0.318 (–0.989, 0.352)	–0.190 (–0.906, 0.527)

BRI, Behavioral Regulation Index; MI, Metacognition Index; GEC, global executive function score. Model 1 is adjusted for age, gender, paternal and maternal educational level, household monthly income, and BMI. Model 2 is further adjusted for moderate-to-vigorous intensity physical activity, and after-school sedentary time plus variables in Model 1. Model 3 is additionally adjusted for other dietary intakes, such as fruits, vegetables, cereals, fish, red meat, fried food, and sugar-sweetened beverages plus variables in Model 2. *p < 0.05, **p < 0.001.

**TABLE 5:** Multivariate linear regression for analyzing associations of milk or yogurt with executive function.

	Milk unstandardized β (95% CI)			Yogurt unstandardized β (95% CI)		
Scale/Index	Model 1	Model 2	Model 3	Model 1	Model 2	Model 3
Inhibit	–0.263 (–0.572, 0.045)	–0.189 (–0.518, 0.140)	–0.130 (–0.479, 0.219)	–0.166 (–0.640, 0.308)	–0.077 (–0.578, 0.423)	–0.160 (–0.696, 0.375)
Shift	–0.459 (–0.771, –0.146)*	–0.427 (–0.762, –0.093)*	–0.390 (–0.745, –0.035)*	–0.433 (–0.912, 0.046)	–0.332 (–0.840, 0.176)	–0.262 (–0.804, 0.280)
Emotional control	–0.365 (–0.662, –0.068)*	–0.271 (–0.590, 0.048)	–0.210 (–0.551, 0.131)	0.079 (–0.379, 0.537)	0.066 (–0.421, 0.552)	0.019 (–0.503, 0.542)
*BRI*	–0.380 (–0.684, –0.077)*	–0.310 (–0.633, 0.014)	–0.237 (–0.580, 0.107)	–0.183 (–0.650, 0.283)	–0.129 (–0.620, 0.363)	–0.160 (–0.686, 0.366)
Initiate	–0.655 (–1.103, –0.298)**	–0.518 (–0.901, –0.135)*	–0.509 (–0.917, –0.101)*	–0.582 (–1.131, –0.033)*	–0.369 (–0.953, 0.215)	–0.107 (–0.731, 0.517)
Working memory	–0.408 (–0.777, –0.040)*	–0.223 (–0.611, 0.166)	–0.168 (–0.581, 0.245)	–0.384 (–0.950, 0.182)	–0.158 (–0.751, 0.435)	–0.028 (–0.661, 0.604)
Plan/Organize	–0.465 (–0.870, –0.060)*	–0.226 (–0.653, 0.202)	–0.169 (–0.621, 0.283)	–0.204 (–0.821, 0.413)	–0.015 (–0.660, 0.631)	0.128 (–0.562, 0.818)
Organization of materials	–0.634 (–1.011, –0.257)**	–0.455 (–0.861, –0.048)*	–0.428 (–0.860, 0.005)	–0.164 (–0.744, 0.416)	0.032 (–0.587, 0.651)	0.024 (–0.639, 0.687)
Monitor	–0.524 (–0.960, –0.088)*	–0.260 (–0.727, 0.207)	–0.150 (–0.647, 0.347)	–0.073 (–0.739, 0.593)	0.084 (–0.625, 0.792)	0.193 (–0.568, 0.955)
*MI*	–0.576 (–0.984, –0.169)*	–0.339 (–0.769, 0.091)	–0.259 (–0.713, 0.195)	–0.239 (–0.858, 0.381)	–0.020 (–0.670, 0.631)	0.108 (–0.585, 0.801)
*GEC (BRI + MI)*	–0.487 (–00.859, –0.115)*	–0.293 (–00.685, 0.099)	–0.212 (–00.626, 0.202)	–0.233 (–00.799, 0.334)	–0.061 (–00.654, 0.532)	–0.011 (–0.643, 0.621)

BRI, Behavioral Regulation Index; MI, Metacognition Index; GEC, global executive function score. Model 1 is adjusted for age, gender, paternal and maternal educational level, household monthly income, and BMI. Model 2 is further adjusted for moderate-to-vigorous intensity physical activity, and after-school sedentary time plus variables in Model 1. Model 3 is additionally adjusted for other dietary intakes, such as fruits, vegetables, cereals, fish, red meat, fried food, and sugar-sweetened beverages plus variables in Model 2. *p < 0.05, **p < 0.001.

## Discussion

Considering different types of dairy intake (full- and low-fat, milk, and yogurt), we provided a comprehensive evaluation of the associations of dairy intake with children’s executive function assessed by BRIEF. Generally, results showed that higher dairy intake is associated with a lower T-score for BRIEF, indicating superior executive function performance. In particular, negative associations were observed between full-fat dairy intake and Initiate and Working Memory, and low-fat dairy intake and Organization of Materials, indicating that a higher intake of dairy, irrespective of fat content, is correlated with superior executive function performance. In addition, a positive relationship between dairy intake and indices of Shift and Initiate only was observed in milk, not in yogurt.

Dairy products have been advertised for a long time as being excellent sources of nutritional components and as a part of a well-balanced diet, but there are still large regional differences in dairy consumption (31). In the present study, the average total dairy intake of children was 180.81 ml/day, which was higher than the average dairy intake of 126.7 g in 2019 (32). The possible reason is that the sample of this study was sampled from Guangzhou, China, which has relatively good economic development. In addition, whether dairy intake is beneficial or detrimental to executive function is controversial (19, 33, 34). In general, our analysis supported that a higher intake of dairy was related to superior executive function in children, which was consistent with several studies (9, 17, 20). Interventional studies on children have reported that increasing the intake of certain dairy products could promote executive function (9), those with higher dairy intake performed better in academics (17), and they scored higher on cognitive tests than those with lower dairy intake (20). Meanwhile, some studies conducted among adults and the elderly found that higher dairy intake is likely to have a protective effect against cognitive impairment, such as Dementia (11, 35). On the contrary, there are some inconsistent reports of associations between dairy intake and executive function (19, 33). No association was observed between dairy intake and cognitive ability assessed by the Wechsler Intelligence Scales in a 6-year longitudinal study (18). Additionally, several studies carried out on older adults suggested that greater dairy intake is related to poorer memory performance and cognitive function (36, 37). The pieces of literature with these inconsistent conclusions pointed out that the failure to distinguish between high- and low-fat dairy intakes is a possible reason (18, 36). Based on the above, the current study explored the role of dairy fat content in this relationship. We observed that intake of full-fat dairy was negatively correlated to Initiate and Working Memory, and low-fat dairy was negatively related to Organization of Materials, indicating that both full- or low-fat dairy higher intake are correlated with superior children’s executive function. Some evidence supported our research conclusions. Moderate intake of milk fat can improve neurodevelopment, which has been confirmed in animal experiments and interventional population studies, as most milk fats are rich in phospholipids and sphingolipids (38). On the other hand, the heterogeneity of the study population may also partially explain the inconsistency of results. The sample population for this study is children aged 6–12, who are at the age of rapid brain development (6, 15). However, the sample population for these studies (36, 39) is adults or elderly people who are in a period of plateau or decline in cognitive function.

Growing evidence indicated that the effects of dairy intake on cardiovascular and metabolic health appear to vary by type (i.e., milk, yogurt, etc.) and not just by fat content (40, 41). Thus, analysis of further types of dairy intake may provide insightful information on how dairy intake modifications likely affect children’s executive function. Indeed, it was determined that associations between dairy intake and children’s executive function may vary depending on the types of milk or yogurt in the current study. Specifically, a significantly positive association exists only in milk with executive function, whereas it disappears in yogurt with executive function. Previous studies conducted in Australia, England, and South Africa reported that sugar content in yogurt is generally higher than that in milk (23). Yogurt was often added with sugar to improve its taste (42). According to the survey, yogurt with 10% sugar content is more popular than those with 7% sugar content (43). The above results highlighted the possibility that those who frequently consume yogurts could be at increased risk of exceeding their recommended daily intake of sugar (23). In particular, studies have confirmed that excessive sugar intake will affect the synaptic connections and neurotransmitter transmission in the developing brain (44). Considering the unfavorable contribution of excessive sugar content to children’s brain development, we speculate that it is a possible reason for the inconsistency in the relationship between the two (milk and yogurt) and children’s executive function performance.

The biological mechanisms linking dairy intake to cognitive function are plausible. Dairy products are rich in β-lactolin, which is a β-lactoglobulin-derived Gly-Thr-Trp-Tyr tetrapeptide (45). A previous study using a mouse model demonstrated that β-lactolin can increase monoamine levels in the cortex and hippocampus regions (46). Moreover, it can also promote spatial working memory and attention in mice with pharmacologically induced amnesia (46). A randomized, double-blind study on humans also reported that supplementation with β-lactolin increases neural activity, as indicated by the P300 amplitude, in the parietal area during auditory tasks that require attention (47). Thus, higher intake of dairy may lead to changes in the brain structure and function, particularly in the frontal cortical regions involved, through synapse formation, in neurogenesis, myelination, and glucose control.

To the best of our knowledge, this is the first study based on a large sample to provide an evaluation of the associations of dairy intake with children’s executive function assessed by BRIEF, a highly reliable tool for measuring capacity for everyday skills. Findings from this novel study contribute to an important foundation, more rigorous investigations in associations between types of dairy intake and children’s executive function to identify potential targets for intervention to improve children’s executive function through diet modification. However, several study shortcomings should be considered in regard to the generalizability of our data. First, a cross-sectional study lacks the ability to demonstrate causality. Second, although this study adjusted for many confounders, we could not entirely exclude some underlying factors, such as intelligence quotient (48), which may mediate the observed associations. Third, our research did not include all types of dairy products, such as cheese, milk-containing beverages, and milk-containing ice cream. Although the children in this study had extremely low intakes of these dairy products, the lack of consideration for them may still affect the stability of the relationship. Fourth, alternatives to cow’s milk intake, such as goat’s milk intake, were not considered in this study, and future research should take them into account.

## Conclusion

This study shows that a higher intake of dairy, irrespective of fat content, is related to better executive function performance among children aged 6–12. In addition, a significantly positive relationship between dairy intake and executive function’s indices of Shift and Initiate only was observed in milk, not in yogurt. This study might contribute to identifying potential targets for intervention to improve children’s executive function through dairy intake modification.

## Data Availability Statement

The raw data supporting the conclusions of this article will be made available by the authors, without undue reservation.

## Ethics Statement

The studies involving human participants were reviewed and approved by the Ethics and Human Subject Committee of Sun Yat-sen University. Written informed consent to participate in this study was provided by the participants’ legal guardian/next of kin.

## Author Contributions

YC and QC designed the experiments. XZ and ZG carried out the experiments. XZ performed the statistical analysis and drafted the manuscript. TS, WY, and QC critically revised the manuscript. LC and ZG provided suggestions in the statistical analysis and revised manuscript. All authors read and approved the final manuscript.

## Conflict of Interest

The authors declare that the research was conducted in the absence of any commercial or financial relationships that could be construed as a potential conflict of interest.

## Publisher’s Note

All claims expressed in this article are solely those of the authors and do not necessarily represent those of their affiliated organizations, or those of the publisher, the editors and the reviewers. Any product that may be evaluated in this article, or claim that may be made by its manufacturer, is not guaranteed or endorsed by the publisher.
