# Supplementary material for: Association Between Dairy Intake and Executive Function in Chinese Children Aged 6–12 Years
Source: Front Nutr. 2022 Jul 11;9:879363. doi: 10.3389/fnut.2022.879363 (PMC9309784; doi:10.3389/fnut.2022.879363)
Supplement: Supplementary file 1 [file Data_Sheet_1.docx]

**Table S1 Description of each indicator of BRIEF scale**

| **Scale/Index** | **Description of indicators** |
| --- | --- |
| Inhibit | Inhibitory control; stop one’s behavior at the right moment. |
| Shift | Switch freely from one situation, activity, or problem to another; solve problems flexibly. |
| Emotional Control | Appropriately regulate behavior based on emotion. |
| **BRI** | Effectively switch cognitive settings and manage emotions through rational inhibitory control. |
| Initiate | Start an activity autonomously. |
| Working Memory | The ability to hold large amounts of data in the mind for the purpose of completing a task. |
| Plan/Organize | Set actionable steps to achieve the goal. |
| Organization of Materials | Work equipment, venues, etc. are arranged in an orderly manner. |
| Monitor | Monitor and manage the entire work process. |
| **MI** | Initiate, plan, organize and maintain actions in a state of good working memory to achieve the goal. |
| **GEC(BRI+MI)** | Overall performance of executive function. |

**Table S2 Multivariate linear regression for analyzing associations of full or low-fat milk with executive function**

|  | Full-fat Milk  Unstandardized *β* (95% CI) | | |  | Low-fat milk  Unstandardized *β* (95% CI) | | |
| --- | --- | --- | --- | --- | --- | --- | --- |
| **Scale/Index** | **Model 1** | **Model 2** | **Model 3** |  | **Model 1** | **Model 2** | **Model 3** |
| Inhibit | -0.177(-0.494,0.140) | -0.094(-0.432,0.243) | -0.061(-0.417,0.294) |  | 0.014(-0.629,0.657) | 0.071(-0.611,0.753) | 0.247(-0.484,0.978) |
| Shift | **-0.372(-0.692,-0.052) ^*^** | **-0.385(-0.727,-0.043) ^*^** | **-0.396(-0.755,-0.036) ^*^** |  | -0.410(-1.061,0.241) | -0.264(-0.957,0.429) | -0.001(-0.745,0.743) |
| Emotional Control | -0.280(-0.585,0.025) | -0.178(-0.506,0.150) | -0.147(-0.494,0.199) |  | -0.261(-0.879,0.357) | -0.324(-0.986,0.338) | -0.191(-0.906,0.524) |
| **BRI** | -0.285(-0.596,0.025) | -0.216(-0.547,0.114) | -0.188(-0.536,0.159) |  | -0.199(-0.832,0.434) | -0.190(-0.861,0.481) | 0.046(-0.674,0.767) |
| Initiate | **-0.523(-0.890,-0.155) ^*^** | **-0.436(-0.830,-0.042) ^*^** | **-0.485(-0.900,-0.069) ^*^** |  | -0.552(-1.293,0.189) | -0.177(-0.969,0.616) | 0.119(-0.734,0.973) |
| Working Memory | -0.229(-0.609,0.150) | -0.041(-0.441,0.359) | -0.055(-0.476,0.366) |  | -0.371(-1.136,0.394) | -0.202(-1.007,0.604) | 0.027(-0.834,0.887) |
| Plan/Organize | -0.305(-0.719,0.110) | -0.081(-0.518,0.357) | -0.064(-0.524,0.396) |  | -0.455(-1.298,0.389) | -0.182(-1.069,0.705) | -0.003(-0.947,0.941) |
| Organization of Materials | **-0.411(-0.798,-0.024) ^*^** | -0.230(-0.647,0.187) | -0.244(-0.684,0.195) |  | **-0.820(-1.601, -0.019) ^*^** | -0.675(-1.513,0.162) | -0.537(-1.437,0.364) |
| Monitor | -0.289(-0.736,0.159) | -0.006(-0.486,0.473) | 0.049(-0.457,0.555) |  | -0.578(-1.479,0.323) | -0.425(-1.387,0.536) | -0.158(-1.192,0.875) |
| **MI** | -0.310(-0.727,0.107) | -0.081(-0.520,0.359) | -0.072(-0.534,0.389) |  | -0.795(-1/641,0.050) | -0.544(-1.437,0.349) | -0.275(-1.219,0.670) |
| **GEC(BRI+MI)** | -0.285(-0.665,0.094) | -0.101(-0.500,0.298) | -0.083(-0.502,0.336) |  | -0.524(-1.301,0.253) | -0.340(-1.157,0.477) | -0.086(-0.952,0.779) |

BRI,Behavioral Regulation Index; MI,Metacognition Index; GEC,global executive function score.

Model 1 was adjusted for age, gender, paternal and maternal educational level, household monthly income, and BMI.

Model 2 was further adjusted for moderate-to-vigorous intensity physical activity, and after-school sedentary time plus variables in Model 1 .

Model 3 was additionally adjusted for other dietary intake, including fruits, vegetables, cereals, fish, red meat, fried food, and sugar-sweetened beverages plus variables in Model 2.

**P* <0 .05, ***P* <0 .001
